# Global Comparison of Warring Groups in 2002–2007: Fatalities from Targeting Civilians vs. Fighting Battles

**DOI:** 10.1371/journal.pone.0023976

**Published:** 2011-09-06

**Authors:** Madelyn Hsiao-Rei Hicks, Uih Ran Lee, Ralph Sundberg, Michael Spagat

**Affiliations:** 1 Health Service and Population Research Department, Institute of Psychiatry, King's College London, London, United Kingdom; 2 Department of Economics, Royal Holloway, University of London, Egham, United Kingdom; 3 Uppsala Conflict Data Program, Department of Peace and Conflict Research, Uppsala University, Uppsala, Sweden; The University of South Wales, Australia

## Abstract

**Background:**

Warring groups that compete to dominate a civilian population confront contending behavioral options: target civilians or battle the enemy. We aimed to describe degrees to which combatant groups concentrated lethal behavior into intentionally targeting civilians as opposed to engaging in battle with opponents in contemporary armed conflict.

**Methodology/Principal Findings:**

We identified all 226 formally organized state and non-state groups (i.e. actors) that engaged in lethal armed conflict during 2002–2007: 43 state and 183 non-state. We summed civilians killed by an actor's intentional targeting with civilians and combatants killed in battles in which the actor was involved for total fatalities associated with each actor, indicating overall scale of armed conflict. We used a *Civilian Targeting Index* (CTI), defined as the proportion of total fatalities caused by intentional targeting of civilians, to measure the concentration of lethal behavior into civilian targeting. We report actor-specific findings and four significant trends: 1.) 61% of all 226 actors (95% CI 55% to 67%) refrained from targeting civilians. 2.) Logistic regression showed actors were more likely to have targeted civilians if conflict duration was three or more years rather than one year. 3.) In the 88 actors that targeted civilians, multiple regressions showed an inverse correlation between CTI values and the total number of fatalities. Conflict duration of three or more years was associated with lower CTI values than conflict duration of one year. 4.) When conflict scale and duration were accounted for, state and non-state actors did not differ. We describe civilian targeting by actors in prolonged conflict. We discuss comparable patterns found in nature and interdisciplinary research.

**Conclusions/Significance:**

Most warring groups in 2002–2007 did not target civilians. Warring groups that targeted civilians in small-scale, brief conflict concentrated more lethal behavior into targeting civilians, and less into battles, than groups in larger-scale, longer conflict.

## Introduction

Warring groups that compete to dominate the territory of a civilian population face contending behavioral options: target the population or battle the enemy. Studies of the intentional targeting of civilians in armed conflict have been limited primarily to datasets on conflicts that involve states (i.e. governments) [e.g. 1], [2], and to studies of genocide or of mass killing defined as over 50,000 deaths over five years [e.g. 3], [4] [5], [6]. More recently developed conflict datasets such as those of the Uppsala Conflict Data Program (UCDP) [7] used in this study, have allowed more complete analyses of the behavior of armed groups in war by encompassing combatant groups involved in low-to-high intensity armed conflicts and by including conflicts between non-state clans, rebel groups and rebel factions [e.g. 5], [6], [8].

Opportunities to increase the understanding of factors affecting civilian targeting can potentially be multiplied by coupling studies of civilian targeting by human actors with informative parallels across disciplines and in nature. For example, national security defenses against terrorism have been informed by examining: competitive adaptation between predator and prey; relationships with symbiotic or pathogenic bacteria; and immune system defenses against pathogens [9]–[11]. Interdisciplinary studies have found the size, organization, and timing of insurgency violence to show patterns similar to those in ecology and financial markets [12], [13]. In the case of civilian targeting, we consider the dynamics of warring groups and the civilian population to be potentially comparable to the dynamics of competing parasitic bacteria and the parasitized host organism or population as described in a number of recent studies [14]–[19]. A civilian population in war can be considered analogous to a parasitized host in that it possesses a finite resource – the disputed territory – that warring groups are competing to dominate and use. Warring groups can be considered analogous to competing parasitic bacteria in that both can focus their limited resources either on attacking the competitor or on attacking the host or civilian population. In this paper, we will discuss our study and its findings in the context of research from the fields of biological sciences, social sciences, and conflict studies, drawing on parallels between the dynamics of cooperation, organization, and violent competition found in nature and dynamics of human armed conflict [9]–[20].

Typically, studies of armed conflict report findings in terms of absolute numbers of casualties (e.g. counts of civilian fatalities from targeting). However, systematic analysis of the proportional effects of weapons and perpetrators on civilians is being increasingly used to expand the scope and interpretation of conflict casualty findings, with direct implications for human rights, public health, and civilian-protective policies in armed conflict [21]–[27]. For example, studies of a single conflict – the Iraq war – have measured the proportions of women and children among civilian fatalities to identify relatively indiscriminate effects from perpetrators' use of various weapons [25], [26], and to identify varying effects of civilian targeting by perpetrators using different forms of armed violence [26]. For studies of combatant groups across armed conflicts on an international scale, a common problem is that combatant groups are typically aggregated together at the country level, or into ‘government’ versus ‘challenger’, despite the fact that many conflicts involve multiple warring parties [28]. The disaggregation of findings to particular combatant groups, as in our study, allows examination of tactics employed at the group-specific level that could otherwise be obscured by dynamics at the conflict level [28], [29].

Our aim in this study was to describe degrees to which combatant groups in contemporary human warfare concentrated lethal behavior into the direct, intentional targeting of civilians as opposed to battling armed opponents. To do this, we analyzed the universe of all 226 formally organized combatant groups that used lethal armed force during the calendar years 2002 to 2007. For brevity, we hereafter use the term ‘actor’ to describe a formally organized group that was actively involved in an armed conflict that resulted in at least 25 fatalities from armed violence in a year (a threshold that includes low-to-high intensity armed conflicts).

Our paper contributes new information to the field of armed conflict studies in the following ways: First, we integrated three datasets so that all state (i.e. government) actors and all non-state (i.e. rebel or clan) actors in armed conflicts globally could be analyzed for fatalities they caused by targeting civilians and for fatalities from battles in which they were involved. Ours is one of few studies [8], [29] that statistically examines relationships between fatalities from civilian targeting and fatalities from battles. Second, we measure fatalities from civilian targeting as a proportion of total direct fatalities from armed conflict. To do this, we use the *Civilian Targeting Index* (CTI), a proportional measure that we introduce in this paper for efficient measurement and communication of degrees to which actors in armed conflict concentrate lethal behavior into the direct, intentional targeting of civilians as opposed to battling armed opponents. Civilian targeting has been prohibited by formalized social norms on a global scale since the 1949 Fourth Geneva Convention, and by subsequent Associated Protocols I and II [24], [30], making CTI outcomes relevant to international humanitarian law and to studies of social aggression and transgression. Third, our data-based attribution of civilian targeting to named, combatant groups uses a consistent methodology to identify the degree to which specific actors exercised restraint vs. committed civilian targeting. Fourth, we analyze the universe of actors participating in a recent period of armed conflict to reveal larger patterns of lethal behavior in armed competition, specifically in regard to civilian targeting, in real-world environments of contemporary warfare. This addresses an identified need for more studies to use empirical data from real societies and natural settings to complement studies of competition, cooperation and conflict based on theoretical and laboratory modeling [31], [32].

## Results

### Civilian Targeting by Specific Actors

**Figure 1:**
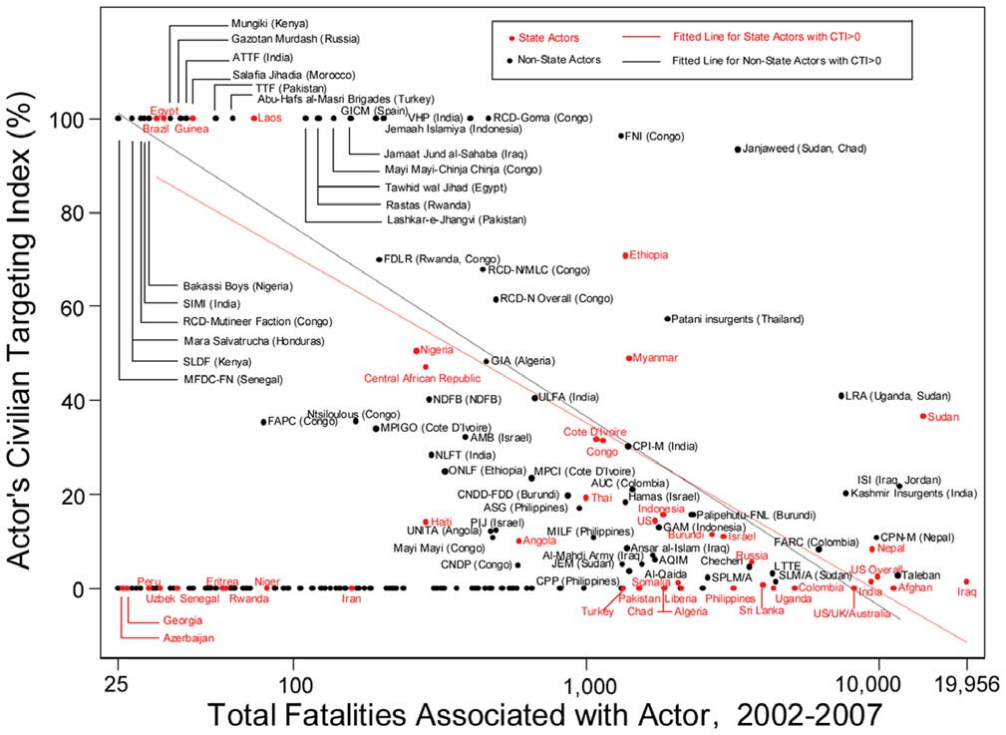
Global comparison of fatalities associated with actors in armed conflict during 2002–2007. Total number of direct fatalities associated with an actor (from battle-deaths and civilian targeting) is plotted against the proportion of total fatalities that was from the actor's civilian targeting, termed the *Civilian Targeting Index* (CTI). Lines show fitted linear regressions for state actors (in red) and non-state actors (in black) that carried out civilian targeting (actor's CTI>0).

Using the Uppsala Conflict Data Program (UCDP) [7], we identified all 226 formally organized armed actors participating in international or civil armed conflicts in 2002–2007: 43 state actors and 183 non-state actors. Our findings for specific actors are shown in Figure 1 and detailed online in Table S1. The x axis of Figure 1 shows ‘total fatalities associated with an actor’ (on logarithmic scale), calculated as the number of civilians the actor killed by direct, intentional targeting plus the number of civilians and combatants killed in battles in which the actor was involved. The y axis of Figure 1 shows the degree to which an actor concentrated lethal behavior into targeting civilians rather than battling opponents in terms of its Civilian Targeting Index. The Civilian Targeting Index (CTI) is the proportion of total fatalities that consists of civilians killed by the actor's intentional targeting (the proportion of total fatalities from battles in which the actor was involved is its reciprocal). In terms of global social norms formalized in laws of war, which are international humanitarian laws and customary standards that delineate the proper treatment of civilians in armed conflict (e.g. the Geneva Conventions) [24], [30], the best possible CTI value is 0 and the worst possible CTI value is 100.

Actors whose CTI values were 100, meaning that 100% of associated fatalities were from their direct targeting of civilians, are found in the upper left quadrant of Figure 1. Our data indicate that actors with CTIs of 100 were all associated with cumulative total fatalities numbering fewer than 500 during the 2002–2007 time period. Nine percent (4/43) of state actors and 11% (21/183) of non-state actors used civilian targeting as their sole form of lethal behavior in conflict (CTI = 100) (*P* = 0.7). Actors whose high rates of civilian targeting contributed to some of the bloodiest conflicts in 2002–2007 are found in the upper right quadrant of Figure 1. For example, the CTI of 96 generated by the non-state Front des Nationalistes et Intégrationnistes (FNI) in the Democratic Republic of the Congo indicates that 96% of fatalities associated with the FNI were unarmed civilians killed by intentional FNI targeting and 4% were combatants or civilians killed in battles between the FNI and an armed opponent. Another non-state group, the Janjaweed, had a CTI of 93: 93% of its associated fatalities were unarmed civilians killed by Janjaweed targeting and 7% were combatants or civilians killed in battles between the Janjaweed and an armed opponent. The state actor Sudan had a CTI of 37 indicating that over one-third of the 14,145 direct fatalities associated with Sudan's government during 2002–2007 were unarmed civilians killed by the government's direct, intentional targeting.

The overall mean CTI for all 226 actors was 18 (95% CI: 13 to 22). Mean CTIs for all state actors (N = 43) and all non-state actors (N = 183) did not differ significantly (Mean state CTI = 19, 95% CI 10 to 29. Mean non-state CTI = 17, 95% CI 12 to 22. *P* = 0.72). Mean CTIs by region did not differ significantly, as suggested by the heavily overlapping 95% CIs shown in Table 1 (*P* = 0.86). The regions that had the greatest numbers of actors in armed conflict were Sub-Saharan Africa (N = 105: 17 state and 88 non-state) and Asia (N = 62: 11 state and 51 non-state).

**Table 1:** Regional Civilian Targeting Index Results: All Actors in Armed Conflict.

Region	Europe			Middle East & North Africa			Asia			Sub-Saharan Africa			Americas		
Actor	All	State	Non-state	All	State	Non-state	All	State	Non-state	All	State	Non-state	All	State	Non-state
**N**	9	3	6	30	6	24	62	11	51	105	17	88	20	6	14
**Mean CTI**	23.35	1.85	34.10	22.39	18.74	23.30	17.29	17.67	17.21	16.99	22.97	15.84	12.89	21.41	9.24
**95% CI**	−10.1 to 56.8	−6.1 to 9.8	−19.5 to 87.7	8.6 to 36.2	−23.3 to 60.8	7.7 to 38.9	9.1 to 25.5	−3.2 to 38.5	7.9 to 26.5	10.8 to 23.2	7.6 to 38.4	8.9 to 22.8	−1.4 to 27.1	−19.7 to 62.5	−6.2 to 24.7
**SD**	43.51	3.21	51.08	36.94	40.04	36.99	32.37	31.06	32.95	32.22	29.96	32.67	30.43	39.13	26.77
**Min CTI**	0	0	0	0	0	0	0	0	0	0	0	0	0	0	0
**Max CTI**	100	6	100	100	100	100	100	100	100	100	100	100	100	100	100
**No. of actors with CTI>0 (%)**	4 (44)	1 (33)	3 (50)	16 (53)	3 (50)	13 (54)	26 (42)	7 (64)	19 (37)	36 (34)	10 (59)	26 (30)	6 (30)	3 (50)	3 (21)
**No. of actors with CTI = 0 (%)**	5 (56)	2 (67)	3 (50)	14 (47)	3 (50)	11 (46)	36 (58)	4 (36)	32 (63)	69 (66)	7 (41)	62 (70)	14 (70)	3 (50)	11 (79)

CTI = Civilian Targeting Index. 226 actors: 43 state and 183 non-state.

### Crossing the Line: Whether Actors used Restraint or Targeted Civilians

Overall, 61% of actors (138/226, 95% CI 55% to 67%) refrained from killing civilians through intentional direct targeting (CTI = 0) and 39% (88/226, 95% CI 33% to 45%) carried out some degree of civilian targeting (CTI>0) during 2002–2007. We used bivariate analysis followed by multivariate analysis of the following variables available in the UCDP datasets of this study to examine for factors associated with actors that used civilian targeting as opposed to restraint: type of actor (state or non-state); scale of armed conflict (in terms of total number of direct associated fatalities); duration of conflict in years; and region of actor.

We first explored relationships between civilian targeting and explanatory variables using bivariate analysis. In absolute numbers, more non-state actors than state actors carried out civilian targeting (64 vs. 24, respectively, with CTI>0). However, a higher proportion of state actors carried out civilian targeting than non-state actors: 56% (24/43) of state actors targeted civilians compared to 35% (64/183) of non-state actors (*P* = 0.012). We considered it possible that the association of state actors with a higher likelihood of targeting civilians was confounded by state involvement in conflicts of greater scale, if scale itself was a factor in whether or not actors targeted civilians, because state actors were associated with a greater mean number of total associated fatalities than non-state actors (State mean = 2,809; 95% CI 1,495 to 4,123. Non-state mean = 708; 95% CI 452 to 963. *P*<0.001). Table 2 shows the distribution of state and non-state actors across varying ranges of total associated fatalities: The largest proportion of state actors (42%, 18/43) was associated with 1,000–4,999 total direct fatalities and the largest proportion of non-state actors (42%, 76/183) was associated with less than 100 total direct fatalities. We also considered it possible that the association of state actors with a higher likelihood of targeting civilians was confounded by state involvement in conflicts of greater duration, if duration was a factor in whether or not actors targeted civilians. For example, proportionally more state actors than non-state actors were engaged in armed conflict for a total of six years: 11/43 (26%) of state actors vs. 17/183 (9%) of non-state actors (*P* = 0.004). Table 3 shows the distribution of actors across different durations of armed conflict. Among the total of 226 actors, 47% participated in armed conflict for one year or less, and 13% participated in armed conflict for the full six years of the study. Regional distributions of actors with no civilian targeting (CTI = 0) and civilian targeting (CTI>0) are shown in Table 1. The region that had the greatest number of actors that targeted civilians was Sub-Saharan Africa (N = 36). However, the proportion of actors that targeted civilians in Sub-Saharan Africa (36/105, 34%) did not differ significantly from proportions of actors of other regions that targeted civilians (*P* = 0.33).

**Table 2:** Distribution of Actors across Ranges of Total Associated Fatalities in 2002–2007.

Range of Total Fatalities Associated with Actor	All Actors (%)	State Actors (%)	Non-state Actors (%)
Over 10,000	5 (2.2)	3 (7.0)	2 (1.1)
5,000–9,999	8 (3.5)	4 (9.3)	4 (2.2)
1,000–4,999	40 (17.7)	18 (41.9)	22 (12.0)
500–999	16 (7.1)	2 (4.7)	14 (7.7)
100–499	69 (30.5)	4 (9.3)	65 (35.5)
Less than 100	88 (38.9)	12 (27.9)	76 (41.5)
Total Actors	226 (100)	43 (100)	183 (100)

**Table 3:** Distribution of Actors across durations of armed conflict in 2002–2007.

Duration of Conflict	All Actors (%)	State Actors (%)	Non-state Actors (%)
**1 year**	107 (47.3)	13 (30.2)	94 (51.4)
**2 years**	37 (16.4)	2 (4.7)	35 (19.1)
**3 years**	27 (11.9)	5 (11.6)	22 (12.0)
**4 years**	12 (5.3)	5 (11.6)	7 (3.8)
**5 years**	14 (6.2)	7 (16.3)	8 (4.4)
**6 years**	29 (12.8)	11 (25.6)	17 (9.3)
**Total**	226 (100)	43 (100)	183 (100)

We then carried out multivariate analysis to analyze for independent contributions to the binary actor outcome of restraint from targeting civilians (CTI = 0) vs. targeting civilians (CTI>0) using combinations of the following explanatory variables: total number of fatalities associated with the actor in 2002–2007 (indicating scale of armed conflict in which the actor was involved); dummy variables for duration of conflict in years (e.g. the variable ‘2 years’ is coded 1 if the actor was involved in conflict for 2 years, 0 otherwise); dummy variables for region of actor; and the dummy variable ‘state’ (equals 1 if state, 0 if non-state). Table 4 shows our logistic regression results. Values in the columns indicate the odds ratio of each explanatory variable. If the odds ratio is greater than 1, the effect on the dependent variable is positive. If the odds ratio is between 0 and 1, the effect on the dependent variable is negative. When duration of conflict dummies were absent (Model 1 and Model 4), the variable for total fatalities was statistically significant, indicating that additional fatalities were associated with increased odds of an actor having targeted civilians. However, with the addition of duration of conflict dummies (Models 2, 3, 5, and 6), the effect of total fatalities became insignificant, with significance dropping from the 99.9% confidence level (*P*<0.001) to the 90% confidence level (*P*<0.1), while coefficients for the duration of conflict had a positive, significant effect on the odds that an actor targeted civilians at some point during armed conflict. For example, in Model 2, the odds that an actor targeted civilians was 3.16 times higher ((3.16−1)×100 = 216%) for an actor involved in 3 years of conflict than for an actor involved in one year of conflict (the comparator duration). The odds that an actor targeted civilians at some point was 7.92 times higher ((7.92−1)×100 = 692%) for an actor involved in 4 years of conflict than for an actor involved in one year of conflict. The significant effect of conflict duration in these models may be because most actors in the one-year duration group (79%, 84/107) had a CTI of 0. The state vs. non-state dummy and the regional dummies never approached statistical significance in these models, suggesting that these actor characteristics had no effect on whether or not actors targeted civilians when other factors were taken into account.

**Table 4:** Logistic regression for independent contributors to actors targeting civilians (CTI>0) as opposed to exercising restraint (CTI = 0).

Explanatory variables	Model 1	Model 2	Model 3	Model 4	Model 5	Model 6
**Total Fatalities**	1.000405****	1.00015	1.000176*	1.000376***	1.000166	1.000188*
**2 years**		1.70			1.72	
**3 years**		3.16**			3.12**	
**4 years**		7.92***			8.59***	
**5 years**		6.01***			7.25***	
**6 years**		5.67***			5.17***	
**3–4 years**			3.41***			3.41***
**5–6 years**			4.65***			4.63***
**MENA**				1.41	.72	.81
**ASIA**				.83	.43	.52
**SSA**				.74	.56	.65
**AMERICAS**				.44	.35	.42
**State**		1.11	1.12	1.48	1.08	1.12
**Number of Actors**	226	226	226	226	226	226
**Pseudo R-square**	.09	.15	.14	.10	.16	.15

*p<0.10.

**p<0.05.

***p<0.01.

****p<0.001.

Dependent variable is 1 if actor CTI>0, 0 if actor CTI = 0.

Values are odds ratios.

In summary, the majority of warring groups (61%, 95% CI 55% to 67%) refrained from intentional, direct civilian targeting during the period of our study. When possible contributors to civilian targeting were examined together in multivariate analysis, a group's involvement in armed conflict for three years or more was associated with an increase in its likelihood of having targeted civilians at some point. These findings do not, however, provide information on factors that may have affected *how much* civilian targeting was carried out by armed groups once they targeted civilians.

### Once the Line is Crossed: Intensity of Civilian Targeting

We examined degrees of civilian targeting by the 88 actors that targeted civilians during 2002–2007, and factors that may have affected how much these actors concentrated lethal force onto targeting civilians as opposed to battling opponents. The mean CTI for all 88 actors that targeted civilians (CTI>0) was 45 (95% CI 37 to 54). There was no statistically significant difference between the mean CTIs of state actors that targeted civilians (N = 24) and non-state actors that targeted civilians (N = 64) (State mean CTI = 35, 95% CI 20 to 49. Non-state mean CTI = 49, 95% CI 39 to 60. *P* = 0.12). Regional analysis of mean CTIs for actors that targeted civilians showed no statistically significant difference by region, as suggested by the overlapping 95% CIs shown in Table 5 (*P* = 0.92).

**Table 5:** Regional Civilian Targeting Index Results for Actors that Targeted Civilians.

	Europe			Middle East & North Africa			Asia			Sub-Saharan Africa			Americas		
	All	S	N	All	S	N	All	S	N	All	S	N	All	S	N
**N**	4	1	3	16	3	13	26	7	19	36	10	26	6	3	3
**Mean CTI**	52.5	5.6	68.2	42.0	37.5	43.0	41.2	27.8	46.2	49.6	39.1	53.6	43.0	42.8	43.1
**95% CI**	−34.7 to 139.7	–	−68.6 to 205.0	19.6 to 64.3	−97.5 to 172.5	18.1 to 68.0	25.4 to 57.0	−5.3 to 60.9	26.9 To 65.5	36.8 to 62.3	17.6 to 60.5	37.4 to 69.8	−3.6 to 89.5	−80.2 to 165.8	−80.3 to 166.5
**SD**	54.8	–	55.1	42.0	54.3	41.3	39.1	35.8	40.0	37.7	30.0	40.1	44.4	49.5	49.7
**Min CTI**	4.6	5.6	4.6	1.5	1.5	5.1	0.7	.7	2.7	1.2	1.2	1.4	8.4	14.1	8.4
**Max CTI**	100	5.6	100	100	100	100	100	100	100	100	100	100	100	100	100

CTI>0 for 88 actors: 24 state (S) and 64 non-state (N).

We fit ordinary least squares linear regressions to actors that targeted civilians to examine whether proportionate levels of civilian targeting (i.e. concentration on civilian targeting) changed with total numbers of associated war fatalities. The total fatalities associated with an actor consisted of civilian fatalities from the actor's direct, deliberate targeting plus civilian and combatant fatalities from battles in which the actor was involved. Because the classical linear model requires the assumption that data have a normal distribution, we tested, and confirmed, that our data for the distribution of CTI's passed normality tests, including: Shapiro-Wilk, Shapiro-Francia, and skewness and kurtosis tests. We applied these tests to all actors, state actors, non-state actors, and to the subsets of these three groups that had CTIs greater than 0, with normality confirmed in all applications. Moreover, we confirmed our linear regression results using non-parametric tests that do not assume normality (available upon request).

A linear regression for all 88 actors that targeted civilians showed a statistically significant correlation for actors associated with greater total numbers of fatalities (i.e. involved in a greater scale of armed conflict) to have caused lower proportions of these fatalities by civilian targeting, with a slope coefficient of −39.1 (95% CI −46.1 to −32.2, t = −11.2, *P*<0.001). We fit separate linear regressions, shown in Figure 1, to state actors and non-state actors that carried out civilian targeting to determine whether they differed in relationships between their degree of civilian targeting and their total associated fatalities. The 24 state actors that targeted civilians had a statistically significant slope coefficient of −35.8 (95% CI −47.0 to −24.5, t = −6.6, *P*<0.001). The 64 non-state actors that targeted civilians had a statistically significant slope coefficient of −40.2 (95% CI −49.3 to −31.2, t = −8.9, *P*<0.001). The difference between the slope coefficients of state actors and non-state actors was not statistically significant, indicating that among actors that targeted civilians, state and non-state actors shared the same quantified dynamic for causing decreasing proportions of civilian-targeted fatalities as they were involved in increasing scales of total armed conflict fatalities. To put it another way, actors that were associated with lower numbers of battle fatalities tended to focus a greater proportion of their lethal behavior onto targeting civilians, with no difference between rebel and government actors.

We then tested whether the finding of decreased concentration on civilian targeting by actors involved in greater scales of conflict held when other explanatory variables were added. Simple linear regressions for the explanatory variable of the log of total fatalities are shown in Model 1 (for all actors with CTI>0), Model 5 (for state actors with CTI>0), and Model 8 (for non-state actors with CTI>0) of Table 6. Table 6 also shows the effect of adding combinations of the following independent variables in ordinary least squares multiple regressions: dummy variables for duration of conflict in years; dummy variables for region of actor; and the dummy variable ‘state’ (equals 1 if state, 0 if non-state). Inclusion of the dummy variables did not improve the goodness of fit of the model, as seen by the adjusted r-square values. In all models for actors that carried out some degree of civilian targeting, the intensity of civilian targeting was unaffected by actors' region or by actors being state vs. non-state. In models for all actors that carried out civilian targeting, duration of conflict in years was a significant factor: actors involved in conflict for three or more years had lower CTI values than actors involved in conflict for one year (the comparator). This was because actors participating in one year of conflict tended to be involved with smaller total numbers of fatalities and to have higher CTI values than actors participating in longer periods of conflict. Finally, and importantly, although the magnitude of the coefficient of logged total fatalities was somewhat decreased when duration of conflict was accounted for, the effect of total fatalities on actors' CTI values remained robust, with a negative direction and high statistical significance.

**Table 6:** Simple and multiple regressions for independent contributors to the degree of civilian targeting (CTI value) of actors that targeted civilians.

	All Actors with CTI>0				State Actors with CTI>0			Non-state actors with CTI>0		
Explanatory variables	Model 1	Model 2	Model 3	Model 4	Model 5	Model 6	Model 7	Model 8	Model 9	Model 10
**Log of total fatalities**	−39.14****(3.49)	−25.53****(6.52)	−39.48****(3.71)	−26.01****(6.66)	−35.76****(5.43)	−26.99**(9.66)	−37.59****(6.15)	−40.24****(4.52)	−25.93***(8.51)	−40.37****(4.70)
**2 years**		−13.04(9.63)		−11.64(10.00)		−57.86**(19.70)			−4.52(11.83)	
**3 years**		−28.84**(11.05)		−28.62**(11.43)		−45.65**(17.85)			−24.10(14.63)	
**4 years**		−36.98**(13.55)		−37.18**(13.98)		−50.56**(19.05)			−33.05*(19.23)	
**5 years**		−30.32**(13.95)		−31.63**(14.56)		−33.30(20.54)			−33.32*(19.19)	
**6 years**		−26.70**(12.92)		−26.30*(13.29)		−19.19(22.71)			−28.42*(16.38)	
**MENA**			−8.40(14.42)	−2.21(14.39)		17.86(19.70)	14.68(25.45)		−9.73(18.32)	−14.75(17.74)
**ASIA**			−3.46(13.87)	3.62(14.00)		26.21(18.92)	10.70(23.49)		−1.52(17.72)	−7.65(17.25)
**SSA**			−7.02(13.60)	−.93(13.76)		32.95*(18.30)	9.96(23.30)		−10.92(17.34)	−12.32(16.85)
**AMERICAS**			−15.35(16.74)	−9.34(16.69)		17.14(21.91)	−6.36(26.29)		−5.94(23.12)	−12.41(22.61)
**STATE**		.57(6.26)	−1.02(6.39)	1.75(6.48)						
**Intercept**	153.87****(10.05)	135.68****(12.99)	161.57****(16.32)	136.92****(18.54)	142.42****(16.92)	121.01****(24.84)	139.65****(31.00)	157.30****(12.56)	142.09****(23.39)	168.52****(19.78)
**Number of Actors**	88	88	88	88	24	24	24	64	64	64
**Adjusted R-square**	.59	.60	.57	.59	.65	.79	.60	.55	.53	53

* *p<0.10*.

** *p<0.05*.

*** *p<0.01*.

**** *p<0.001*.

*Includes actors with CTI>0. Actors with CTI = 0 are excluded*.

*Standard errors in parentheses*.

### Civilian Targeting by Actors in Prolonged Armed Conflict

**Figure 2:**
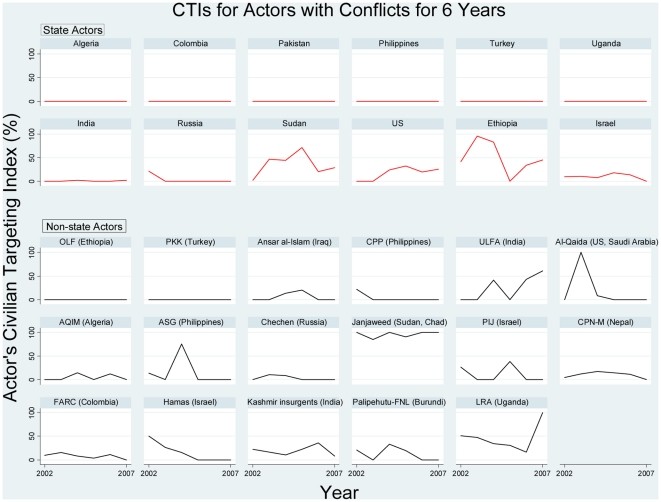
Annual Civilian Targeting Index values for the 29 actors in prolonged armed conflict during 2002–2007.

We analyzed civilian targeting by actors that were involved in prolonged armed conflict for the maximum duration covered by our dataset: six years. Figure 2 shows annual CTI values for the 29 actors in prolonged armed conflict. We included the U.S. because it was involved in armed conflict for six years in total: as a joint actor with the U.K. and Australia against Iraq in 2003, and as an individual actor during the five years of 2002 and 2004–2007 in Afghanistan, Iraq, Pakistan (involving U.S. drone attacks), and Saudi Arabia (in attacks on, and by, representatives of the U.S.). As shown in Figure 2, eight actors refrained from any intentional, direct targeting of civilians throughout prolonged conflict, maintaining a CTI of 0. Twenty-one actors targeted civilians in at least one of the six years.

We analyzed for factors that influenced whether or not actors crossed the line into civilian targeting over the course of prolonged conflict. Because our data included actors' CTIs over a series of six years, we transformed the data into a panel structure for panel data analysis, which confers regression analysis with the capacity to examine cross-sectional data (e.g. on actors' behavior) over time. Table 7 shows our random effect logit regressions for independent contributions to the binary dependent variable of an actor targeting civilians (CTI>0), as opposed to exercising restraint from targeting civilians (CTI = 0). We analyzed using combinations of the following explanatory variables: ‘year’ to identify the time trend; total fatalities associated with the actor within the year (indicating scale of armed conflict within the year); dummy variables for region; and the dummy variable ‘state’ (equals 1 if state, 0 if non-state). We confirmed our random effect logit regression results using random effect probit regressions for robustness checks and confirmed that the direction and the significance of coefficients for each variable remained the same (available upon request).

**Table 7:** Random effect logit regression for independent contributors to actors in prolonged conflict targeting civilians (CTI>0) as opposed to exercising restraint (CTI = 0) during six years.

	All Actors in Prolonged Conflict			Actors in Prolonged Conflict with CTI>0 in at least one year		
Explanatory variables	Model 1	Model 2	Model 3	Model 4	Model 5	Model 6
**Year**	.7595*	.7528*	.7563*	.7603*	.7625*	.7637*
**Total Fatalities**		.9999	1.0000		1.0000	1.0000
**MENA**			.6961			2.8033
**ASIA**			1.5726			4.0489
**SSA**			9.1012			27.9962**
**AMERICAS**			4.4404			11.2273*
**State**			.1699			1.1829
**Number of Observations (actors×6 years)**	174	174	174	126	126	126
**Number of Actors**	29	29	29	21	21	21

*p<0.05.

**p<0.01.

Dependent variable is 1 if actor CTI>0, 0 if actor CTI = 0.

Values are odds ratios.

Models 1 to 3 of Table 7 show results for all 29 actors involved in prolonged armed conflict. Model 1 is a simple regression model that contains the time variable (year) as a single explanatory variable. The odds ratio (.7595) implies that each additional year was associated with a decrease in the odds of targeting civilians of 24.05% ((1−.7595)×100 = 24.05%). For Model 2 and Model 3, we extended Model 1 by including total fatalities within the year, the 4 region dummies, and the state dummy. The following variables had no significant effect on whether an actor targeted civilians vs. exercised restraint: total fatalities within a year, the actor's region, or being a state vs. non-state actor. The time variable, however, remained significant. Holding all other factors fixed, each additional year was associated with a decrease in the odds of targeting civilians of about 24%. Models 4 to 6 of Table 7 focus on the 21 actors in prolonged conflict that targeted civilians in at least one year: for these actors, the time effect continued to be robust, with similarly decreased odds of targeting civilians with each additional year. However, regional effects of actors from Sub-Saharan Africa and the Americas became significantly associated with increased odds of targeting civilians in this subgroup of actors.

We next analyzed for factors that affected the degree to which actors in prolonged conflict targeted civilians. Figure 2 gives the impression that there was no prevailing pattern for increased or decreased civilian targeting over time. We used the random effects model of panel regression because Hausman test results (unreported) indicated that this was a consistent, more efficient model for our data. Table 8 shows panel regressions for relationships between the continuous, dependent variable of an actor's CTI and explanatory variables of: the common log of total fatalities within a given year; time dummy variables D2003 to D2007 to identify a specific year effect (e.g. D2003 equals 1 if the observation is from 2003, 0 if otherwise); dummy variables for region; and the dummy variable ‘state’. The only statistically significant variable was the SSA dummy, indicating that the CTI values of actors fighting prolonged conflicts in Sub-Saharan African countries were higher than those fighting prolonged conflicts in Europe (the comparator region). We found no statistically significant tendency for actors in prolonged conflict to increase or decrease their degree of civilian targeting over time, with total fatalities within a given year, or with state vs. non-state classification of the actor, even when actors that never targeted civilians were excluded from the analysis.

**Table 8:** Panel Regression for independent contributors to the degree of civilian targeting (CTI value) by actors in prolonged conflict during six years.

Explanatory variables	All Actors in Prolonged Conflict	Actors in Prolonged Conflict with CTI>0 in at least one year
**Log of total fatalities**	1.19(3.45)	−2.12(4.66)
**Y2003**	2.43(4.27)	3.52(5.89)
**Y2004**	4.93(4.28)	7.24(5.92)
**Y2005**	−1.04(4.29)	−1.98(5.94)
**Y2006**	−2.52(4.30)	−4.10(5.95)
**Y2007**	−.57(4.36)	−1.97(6.13)
**MENA**	2.86(14.43)	5.07(14.38)
**ASIA**	4.86(14.52)	7.24(14.29)
**SSA**	30.58*(14.72)	44.11**(14.36)
**AMERICAS**	6.18(16.77)	9.40(17.14)
**STATE**	8.42(7.09)	4.19(8.78)
**Intercept**	−4.54(16.89)	6.56(19.04)
**Number of Actors**	29	21
**Number of Observations** **(Actors×6 years)**	174	126
**Wald chi-square**	17.17	23.47
**P-value of Wald chi-square**	.10	.02

*p<0.05.

**p<0.01.

*Standard errors in parentheses*.

In summary, our findings on the 29 actors involved in prolonged conflict indicate that these actors were more likely to completely refrain from civilian targeting (i.e. to have CTI = 0) in later years of conflict than in earlier years. However, their degree of concentration of lethal behavior into targeting civilians as opposed to battling opponents (i.e. their actual CTI value) showed no overall pattern of decrease or increase over time, due to high variability in the behavior of specific actors over the course of prolonged conflict.

## Discussion

Our study shows the degree to which specific, formally organized actors in armed conflict concentrated their lethal behavior into intentionally targeting civilians as opposed to engaging in battles during 2002–2007. We found four significant behavioral patterns in contemporary warfare. First, the majority (61%) of all formally organized actors in armed conflict during 2002–2007 refrained from killing civilians in deliberate, direct targeting. Compared to our finding, a study of actors in interstate wars during 1900–2003 found that just under half refrained from killing civilians in targeting [2]. This study's methodology differed from ours by excluding actors in intrastate conflicts (e.g. civil wars), by including indirect (nonviolent) deaths and by requiring at least 1,000 fatalities per year for inclusion (we require at least 25 fatalities per year for inclusion). We expect that if the study included low-intensity conflicts and intrastate conflicts involving non-state actors, the percentage of actors refraining from civilian targeting would be closer to ours, as we show (in Table 3) that state actors tend to be involved in conflicts of longer duration, which is itself associated with a greater likelihood of carrying out some degree of civilian targeting. This takes us to our next point.

Second, controlling for other variables, actors were more likely to have carried out some degree of civilian targeting, as opposed to none, if they participated in armed conflict for three or more years rather than for one year. In regard to this finding, we speculate that the longer the duration, the more likely that at least some combatants in an actor's armed forces will at some point carry out civilian targeting, which would move the actor from the ‘restraint’ (CTI = 0) to the ‘targeting’ (CTI>0) category. Three possible reasons for an actor's movement from ‘restraint’ to ‘targeting’ categories include: 1.) The actor does not control troops adequately for complete enforcement of a culture of restraint from targeting civilians – complete enforcement requires an increasing expenditure of resources to prevent civilian targeting as the actor has more troops to control for a longer time; 2.) The actor has a combat culture of disregard for civilians and expends no resources on preventing civilian targeting; and 3.) The actor channels resources into a strategy of targeting civilians. Because of the multiple, in some cases nonspecific, factors that can contribute to an actor carrying out some degree of civilian targeting as opposed to none, use in quantitative conflict studies of a binary outcome of civilian targeting vs. no civilian targeting might not be highly productive in examining contributors to civilian targeting. However, because maintaining a CTI of 0 indicates ongoing resource expenditure, and a more specific, nonrandom element of choice, concentrating quantitative and qualitative research on actors that refrain from civilian targeting in war may identify promising avenues for increasing or supporting civilian-protective behavior in war.

Our third main finding focuses on the actors that targeted civilians rather than maintaining restraint from civilian targeting. Once actors targeted civilians, what were the factors that affected the degree to which they concentrated lethal behavior into intentionally targeting civilians? In both simple and multiple regressions, we found that among actors that targeted civilians, those that engaged in greater scales of armed conflict concentrated less of their lethal behavior into civilian targeting and more into involvement with battle fatalities. Conversely, those that engaged in lesser scales of armed conflict concentrated more of their lethal behavior into civilian targeting and less into involvement with battle fatalities. Also, among actors that targeted civilians, those that were involved in conflict for total durations of three or more years concentrated less of their lethal behavior into civilian targeting than those involved in conflict for one year or less. This was because the actors that targeted civilians during one year or less of conflict tended to be involved with smaller total numbers of fatalities and to have higher CTI values than actors participating in longer periods of conflict. These findings suggest that warring groups that targeted civilians during small-scale conflicts of brief duration tended to concentrate more of their lethal behavior into targeting civilians than warring groups that targeted civilians during larger-scale conflicts of moderate or long duration.

Fourth, when factors of scale of conflict and duration of conflict were accounted for, an actor's likelihood and degree of targeting civilians was unaffected by whether it was a state or a non-state group. The absolute number of non-state (rebel) actors that targeted civilians (n = 64) was higher than the number of state actors that targeted civilians (n = 24) only because more non-state actors than state actors participated in armed conflict (183 vs. 43, respectively).

We also examined civilian targeting over the course of consecutive years in the subset of 29 actors that were involved in prolonged conflict of six years duration in 2002–2007. Controlling for other variables in panel data analysis to examine cross-sectional data on actors' CTIs over time, we found that actors in prolonged conflict were more likely to refrain from civilian targeting (with a CTI = 0) in later years of conflict than in early years. Nevertheless, for actors in protracted conflict, their degree of concentration on targeting civilians as opposed to battling opponents (i.e. their actual CTI value) showed no overall pattern of decrease or increase over time, due to high variability in the behavior of specific actors over the course of prolonged conflict. The only clear association was that CTI values for sub-Saharan African actors tended to be higher than for other regions. In earlier analyses of all 226 actors, we analyzed for the variable of ‘total fatalities summed for all years of conflict’. This variable was not examined for association with actors' CTI values tracked over consecutive years of prolonged conflict, since it lacks the time-specific element. Our analyses for ‘all actors’ and for ‘actors in prolonged conflict’ examine different ‘total fatality-time’ dynamics. For actors in prolonged conflict we used the fatality measure of ‘total fatalities within the given year’. There was no evidence for this subset of 29 actors in prolonged conflict that there was any association between high total fatalities within a given year of conflict and their CTI value for that year, although our failure to find this could be a consequence of small sample size and numerous explanatory variables. Analysis for longer, or different, periods of time than our study could show different results.

As our findings show, combatants' adherence to global social norms against targeting civilians can be quantified to identify the worst offenders in contemporary warfare, to show variance between actors, and to identify broad patterns of human behavior in armed conflict. Civilian Targeting Index (CTI) outcomes that measure the proportional degree to which actors concentrate lethal behavior into targeting civilians may be more informative than binary outcomes that indicate targeting vs. restraint for indicating probable cases of systematic, strategic civilian targeting. Actors whose total fatalities from armed conflict were caused in large part by their intentional targeting of civilians, as indicated by high CTI values in our paper, can be considered more likely to have used civilian targeting as a deliberate, systematic strategy in armed conflict, especially if associated with high absolute numbers of fatalities [24], [25].

Although we refer to a variety of studies across disciplines to discuss our findings, a particular, though rough, analogy can be made between a form of microbial warfare and our findings on human warfare. Many types of bacteria use chemical weapons when fighting in competition against other bacteria to parasitize a host, some releasing their bacteriocins (bacteriocidal toxins) by suicidal self-explosion to kill competitors [14]–[19]. This is an example of ‘spiteful behavior’ in nature, which is harmful to both the actor (e.g. the bacterial suicide attacker) and the recipient (e.g. the targeted bacterial opponent) [14]–[17]. A parasitic bacteria's harm to the host is ‘selfish behavior’, being beneficial to the actor (e.g. the parasite) and harmful to the recipient (e.g. the weakened or killed host) [15]. A civilian population in war is comparable to a parasitized host in that it possesses a finite resource - the disputed territory - that opposing actors are competing to dominate and use. Warring actors can attempt to shift the dynamics of this competition in their favor by focusing their energies onto controlling or eliminating the civilian population, or on controlling or eliminating their opponent, by lethal force. In addition to competing for territory, armed groups compete, sometimes using lethal coercion, to gain other resources of the civilian population: food, information, logistical support and political support. We believe that our study's finding that warring actors concentrate less on killing civilians if they are involved in more lethal battles against armed opponents is analogous to the decreasing virulence to host organisms found as competing parasitic bacteria kill each other more in direct battles using bacteriocins [14]–[16], [18], [19].

Cooperative behavior exists at many levels in nature [31] and has been shown to be increased by enforcement through punishment, policing and sanctions in humans, meerkats, fish, social insects, bacteria and plants [31], [32], [33]–[36]. One of the best-known examples of cooperation in humans is warfare, in which soldiers place themselves at risk of injury or death in an activity that benefits others [33], [36] (analogous to the ‘spiteful’ behavior of bacteriocin-producing bacteria [14]–[17]). Once actors are at war, the exercise of restraint to comply with global social norms (e.g. laws of war) requires an additional level of cooperation. For example, in an asymmetric, irregularly-fought war in which Side A soldiers disguise themselves as civilians, a Side B soldier could likely decrease his or her individual risk by killing all those encountered who look like civilians. Not only do Side B soldiers place themselves at risk by directly battling Side A soldiers, they accept additional risk when they do not target the civilian population that could include or support hidden Side A soldiers. In our study, it is probable that higher levels of cooperation, resources and maintenance of discipline (i.e. enforcement) were required to ensure that all soldiers of a combatant group refrained from targeting civilians to result in actors with CTIs of 0.

On a social level, it may be that actors that refrain from civilian targeting are responding to historically recent global social norms that prohibit the targeting of civilians, formalized in treaties and customary standards that constitute contemporary laws of war [24], [37], [38], whereas the regression lines in Figure 1 represent trends in lethal behavior of actors that operate according to cost-benefit considerations in which cooperation with, or punishments against breaching, global norms against civilian targeting have, or are considered to have, little effect on the actor's success. It would be of interest to examine whether the percentage of actors that refrain from civilian targeting, and the regression slope for actors that carry out civilian targeting (Figure 1), are different for conflicts fought before and after the creation of international norms against civilian targeting such as the Geneva Conventions of 1949. Replication studies using comparable inclusion criteria and extending beyond our study's timeframe will be valuable to test our findings, as we only show actors' civilian targeting during 2002–2007, based on the UCDP data available at the time of our study.

The proportion of fatalities caused by civilian targeting may be affected by different factors and dynamics than those affecting the absolute number of civilians killed in targeting. For example, although studies of state actors have suggested that longer duration of conflict is associated with actors killing greater absolute numbers of civilians [1], [2], this is compatible with findings from our study, which differs by focusing on the *proportion* of total fatalities caused by civilian targeting in order to quantify an actor's concentration of its efforts into civilian targeting as opposed to engaging in battles. Absolute numbers of civilians killed by targeting can be calculated from our data by applying the actor's CTI value (a proportion) to the total fatalities associated with the actor. However, we believe that a distinctive value of our study is its exposure of behavioral patterns of targeting civilians in war through a focus on proportional analyses.

Other studies in the fields of social sciences, natural sciences, and conflict studies suggest that the following additional variables will be important to examine in future research on the dynamics of groups' concentration on civilian targeting vs. battling opponents: regime type [1], [5], [8]; spatial distribution [14], [16], [19]; actors' reasons, costs and resources for war [1], [2], [8], [29], [39]–[41]; degree of relatedness between opposing actors and between actors and civilians [11], [14], [15], [18], [39]; and behavior of the civilian population [9], [11], [41]. Civilian populations may tolerate or mount resistance against use of their resources or territory by warring actors and may do so in complex ways that vary with actors and their circumstances [41], similar to a parasitized host immune system interacting with, or reacting against, pathogens [9], [11].

Eck and Hultman [5], who also use the UCDP one-sided violence dataset, find that the regime type of the country in which actors target civilians is associated with numbers of civilians killed by targeting, with higher numbers of targeted civilian fatalities in autocratic and democratic countries and lower numbers in semi-democracies. This pattern is driven by autocratic state actors killing greater numbers of civilians by targeting within their countries and by non-state actors killing greater numbers of civilians by targeting in democratic countries [5], [8]. Findings from studies that are limited to mass killings and genocide [e.g. 3], [4]; that exclude actors involved in non-state conflicts [e.g. 1], [2]; that combine direct and indirect deaths [e.g. 1], [2]; or that combine civilian fatalities from both targeted and indiscriminate violence [e.g. 1] may be suggestive but are not directly applicable to this and other studies that examine direct, targeted fatalities from violence of low-to-high intensity involving all conflict actors [5], [6], [8].

Actors' reasons, costs or resources for war can affect civilian targeting [1], [2], [8], [29], [39]–[41]. Actors' resources in war can include numbers and effective capacity of soldiers; numbers and effective capacity of weapons; financial resources; political power; control of territory; and civilian support. The dynamics of civilian targeting can be affected by both absolute and relative resources of actors in a conflict. For example, Vargas finds empirical support from data on the Colombian civil war for his model predicting that an actor that comes into power kills more civilians in territories where its enemy is powerful, possibly to coerce a shift in civilian support [40]. Vargas's study is one of many that address the proposal by Kalyvas [41] that actors in civil wars target civilians as a group (which he calls ‘indiscriminate’ violence) more in territories that are controlled solidly by their opponent and that actors use personalized targeting of individual civilians (called ‘selective’ violence) more in territories where they have partial but not complete control, in order to shift civilian support from opponents.

Rather than focusing on where actors target civilians based on relative control over territory [41], Hultman focuses on when actors target civilians, and how many they kill, based on their strength relative to armed opponents on the battlefield [29]. Her study of civilian targeting by 60 rebel (non-state) actors over 2002–2004, showed that rebels killed greater numbers of civilians in targeting after losing more rebel fighters in battles, and after killing fewer government (state) fighters in battles. In a similar study of 212 non-state groups in conflict with state actors in 1989–2004, Wood [8] measured relative strength of opposing actors as the ratio of numbers of rebel troops to government troops and found that weaker rebel actors, relative to their government opponents, killed higher numbers of civilians by targeting, with an additional effect that weaker rebels further increased civilian targeting if the state actor also targeted civilians. Although civilian targeting by state actors was not measured as an outcome in these studies [8], [29], their primary finding, which Hultman summarizes as “rebel violence against civilians is, like terrorism, the weapon of the weak” [29, p. 218], relates closely to our finding that the less that actors were associated with battlefield fatalities, and the shorter they fought, the more that they concentrated lethal force onto targeting civilians; a finding that could be consistent with the explanation of battlefield weakness of actors. Although our findings show that state and non-state actors had the same statistical relationship between concentration on civilian targeting and total conflict fatalities, further research is needed to determine whether battlefield weakness can explain high concentrations of civilian targeting by state actors.

Hultman speculates that weak rebels target civilians as an alternative strategy to fighting battles because it is a relatively cheap and easy way to impose extra political and military costs on its state opponent, and in order to signal the rebel's power and the state's impotence in settings off the battlefield [29], [39]. The signaling function of civilian targeting by weak rebel actors has been described in anthropological research on civilian targeting by rebels in Sierra Leone and Liberia [42]. As a Sierra Leonean commander summarized:

That [targeting civilians] is one of the major tools in guerrilla warfare. Because when the guerrilla is fighting, he is less equipped, he has less manpower. He's going to use tactics to put fear into the civilian populace and send the signal to the government that it can't protect its people…It is one of the tools the guerrilla uses. Fear and intimidation. [42, p. 222]

Human actors are particularly able to fine-tune cooperative behaviors (e.g. warfare) quickly in response to proximate factors affecting the direct benefit of cooperation during competition at local and global levels [35]. Local cultural constructions regarding the nature of political power have been described as predominant factors in non-state actors' civilian targeting, even while simultaneously these actors vie for political and symbolic power in the global context of armed conflicts by using the global media [42]. International research shows wide variation in local social norms for cooperation, punishment and response to punishment across societies with different cultures, social histories and strength of rule of law [36]. Although much of the research we describe, including our own, points to broad patterns of behavior regarding targeting civilians, local contexts of meaning (e.g. what is ‘power’ or ‘success’ in a conflict) may interact with global social norms to affect the behavior of specific human actors [36], [42]; affecting social norms, costs and benefits within the context where tactics are used, and affecting whether actors depart from general trends to become outliers with unusually low or unusually high levels of concentrating lethal behavior into the deliberate targeting of civilians during armed conflict.

## Materials and Methods

To create the dataset used for our study, in which all formally organized state and non-state actors participating in international and civil armed conflicts are represented, we combined three datasets compiled by the Uppsala Conflict Data Program (UCDP) [7] for their overlapping periods of 2002–2007: the UCDP One-Sided Violence Dataset v. 1.3 1989–2007 for civilian targeting by state and non-state actors [43], [44], the UCDP Battle-Related Deaths Dataset v. 5 2002–2007 for fatalities from battles involving at least one state actor [45], [46], and the UCDP Non-State Conflict Dataset v. 2.1 2002–2007 for battle-related deaths from battles between two non-state actors [47], [48]. Our data describe actors that were associated with at least 25 fatalities, as UCDP requires a minimum of 25 fatalities in a year for an actor to be included in a UCDP dataset; a low threshold that allow inclusion of the low-intensity conflicts in our data. In regard to civilian targeting specifically, the inclusion of low-intensity conflict is in contrast to datasets that predated the UCDP one-sided violence dataset and included only mass killings or genocide [5].

UCDP produces ‘Best’, ‘Low’ and ‘High’ estimates of deaths based on assessment by human coders of data from a wide range of open-source, independent sources: the media, NGOs (non-governmental organizations), governments, international agencies, truth commissions, and academic reports. Best estimates are based on UCDP coders' evaluation of the sources' credibility and tend to be conservative [5], [49]. We used UCDP Best estimates to provide a systematically derived baseline estimate of fatalities. This baseline is expected to undercount deaths to some degree because some deaths will always go unreported [5], [49]. To date, systematic studies have not been done to determine if civilians killed by targeting are any more, or less, likely to have their fatalities included in the UCDP data than fatalities of civilians and combatants killed in battles, which would be the kind of bias that could affect our proportional CTI analysis. We chose to use UCDP Best estimates because they are considered to provide a confident lower bound for the analysis of trends [5], [49], for which conservative and consistent coding practices are critical, and because they are used in key, relevant UCDP data analyses in the literature [5], [6].

What we call ‘civilian targeting’ in our paper is termed ‘one-sided violence’ by UCDP [43], [44], and is defined as the direct and intentional (also called deliberate) killing of civilians by use of armed force [5]. UCDP's one-sided violence includes acts such as genocide, terrorist attacks on civilians (but not on government or military targets), mass executions and individual extrajudicial executions (except for extrajudicial killings in a government prison or facility). One-sided violence does *not* include indirect deaths from conflict, unintentional (also called ‘collateral’) civilian deaths, or deaths from disregard for civilians when actors attack each other (e.g. in indiscriminate violence during battles). Our analysis includes only formally organized armed groups because the available version of the UCDP One-Sided Violence Dataset excluded violence by loosely organized groups such as some clans, tribes and ethnic groups [44].

We calculated ‘total fatalities associated with an actor’ as all UCDP's ‘one-sided violence’ fatalities by the actor [5], [43], [44], [49], plus all UCDP ‘battle-deaths’ from battles involving a state actor in which the actor was involved [45], [46], [49] plus all battle deaths from battles involving only non-state actors in which the actor was involved [47], [48]. In simpler terms, we calculated ‘total fatalities associated with an actor’ as the number of civilians the actor killed by direct, deliberate targeting plus the number of civilians and combatants killed in battles involving the actor. UCDP ‘battle-deaths’ are associated with each actor involved in the battle and combine civilian and military fatalities in battle because many battle data do not attribute deaths to specific actors or distinguish civilian from combatant deaths. UCDP battle-related deaths are all fatalities – military and civilian – directly related to combat between two military actors [46], [49]. Battle-related deaths include fatalities from traditional battlefield fighting; from guerrilla activities such as hit-and-run attacks or ambushes; and from bombardments of military bases, cities or villages: as long as the intended targets are either military actors or representatives of the actors.

UCDP battle-related deaths include both indiscriminate and unintentional (‘collateral’) deaths of civilians. The killing of civilians in indiscriminate warfare, in which actors do not distinguish between civilians and opponent combatants, is a form of lethal behavior which is distinct from the targeting of civilians, but which is also prohibited under international humanitarian laws and customary standards [24], [30]. Both indiscriminate and unintentional deaths of civilians are important on moral and social grounds, and can have substantial quantitative impact in terms of fatalities. An actor could refrain from intentionally targeting civilians, yet exact an unacceptably high toll on civilians in terms of the absolute number or proportion of civilian deaths among battle deaths. Other studies would be needed to examine the dynamics of actors inflicting indiscriminate or unintentional civilian fatalities, which are difficult to distinguish in practice in compiling conflict data, and which may differ from the dynamics we find for civilian targeting.

We calculated the ‘Civilian Targeting Index’ as a proportion: the number of civilians killed in direct targeting by the actor, divided by the total fatalities associated with the actor. To the extent that battle-deaths constitute the total associated fatalities of an actor, total associated fatalities of an actor overlap with total associated fatalities of other actors involved in those battles. This does not, however, confound our CTI findings, which are civilians killed by targeting as a *proportion* of the total fatalities associated with an actor.

We show the following data for each of the 226 specific actors online in Supplementary Table S1: Actor name; Civilian Targeting Index (CTI); rank by CTI from worst (highest CTI = 100) to best (lowest CTI = 0), total associated fatalities, and rank by total associated fatalities. Actors are identified in the dataset more than once if they acted alone and jointly. For example, the US is shown as a sole actor and as a joint actor with the UK and Australia in Iraq in 2003. Due to UCDP coding procedures established before the period of this study, there are three actors whose involvement in fatalities is recorded under partner actors when acting in cooperation: ‘Janjaweed’ results are for the Janjaweed acting alone, while the Janjaweed acting with the Sudanese government is coded under ‘Sudan’. ‘US’ results are for the US acting alone, while the US acting with Iraq or Afghanistan governments is coded under ‘Iraq’ or ‘Afghanistan’, respectively. ‘US/UK/Australia’ results are for US/UK/Australia acting alone, while US/UK/Australia acting with Iraq's government is coded under ‘Iraq’.

Fatalities are not included in the UCDP conflict dataset if they cannot be associated with any actor (e.g. dead bodies recovered on a street). This stringent requirement of the UCDP coding process means that civilian targeting findings from our dataset can be understood to reflect civilian targeting by combatant groups only, without inclusion of fatalities resulting from criminal activity from noncombatants in the conflict environment. Because the perpetrator of civilian targeting must be identified in order for the fatality to be included in the UCDP one-sided violence dataset [6], specific counts of numbers of fatalities from civilian targeting derived from our data should be considered with caution, as they lack the robustness of the broad, proportional trends that we present in our findings. Our data describe actors associated with conflict fatalities during 2002–2007 only: Civilian targeting findings for specific actors could differ substantially depending on the time period covered.

The UCDP's data collection methodology of relying on secondary sources (the media, NGOs, governments, international agencies, and academic reports) for information on violent fatalities has the potential to introduce biases arising from how these sources gather and publish their information. Kalyvas has described [41] how partisan bias and various forms of urban bias can affect fatality reporting by all these types of sources. However, studies that examine conflict coverage bias using substantial datasets have been few, and older studies of media coverage of violence cannot reflect technological advances that have changed data-gathering capacities for recent armed conflicts. One study found that international news articles covering civil wars in 1992–1999 very slightly increased as conflict intensity increased, but at the most extreme intensity of conflict (over 20,000 casualties per month), such as was only present in the Rwandan civil war during the study, the number of news articles covering the conflict started to decrease, possibly due to the poor quality of information filtering out of Rwanda at the height of the genocide [50]. A study that compared UCDP battle-death data for 1989–2002 to fatality data from other sources suggested that the predominance of English-language sources in UCDP searches led to good coverage of fatalities in the Northern Ireland conflict, but undercounted fatalities in Spanish-speaking Colombia's civil war (although UCDP trends over time generally matched well) [51]. An exceptionally wide gap occurred between UCDP fatality numbers and locally-sourced Colombian fatality numbers in a year that was marked by particularly intense conflict coupled with Colombia's pivotal presidential election. The authors speculated that in an overload of internationally newsworthy stories from Colombia, many smaller conflict events (and their associated fatalities) were not picked up by international news agencies [51].

UCDP spends almost equal time collecting data from news media and from NGO reports, monographs, and other sources. UCDP then triangulates between multiple sources to estimate actors' fatality figures (e.g. witness reports to a truth commission may supplement or be compared to media reported data and NGO reports on a massacre). Reports are traced back to their primary source, when possible, in order to determine reliability, and potential biases of sources are taken into consideration when determining UCDP Best estimates [5]. UCDP includes local news reports in its searches to some extent [5], but is limited to reports published or translated into the English language. Journalistic coverage of some areas, such as sub-Saharan Africa, may be lower, making it difficult to establish exact numbers of fatalities [5], [49]. Although UCDP fatality numbers can be viewed as being “too low”, i.e. not perfectly representing the actual number of fatalities from a conflict or from one-sided violence, UCDP does not claim to provide a perfect mirror-image of reality, but instead stresses that its Best estimates provide a systematically derived, reliable baseline, useful for cross-country and temporal comparison [5], [49].

Although we have described here the limitations and possible biases that can affect UCDP estimates of absolute numbers of fatalities, it is important to emphasize that no published critique has questioned or tested civilian targeting to battle-death fatality ratios of the kind we use in our study. Plausible critiques that are relevant to our study could include that some actors are better at hiding their hand in massacres than are others (thus lowering their CTI), or that there are large undercounts for total deaths specifically for actors with high CTI scores. To date these possible biases have not been systematically studied. Although these potential biases should be kept in mind by the reader, especially when viewing findings for specific actors, we know of no clear reason to believe that these possible problems are of a magnitude and consistency that would compromise the global trends we find in our study.

Stata 11.1 was used for statistical analysis to calculate means, proportions and regressions. Proportions were compared using chi-square testing, and means using one-way ANOVA, to obtain two-tailed P values.

## Supporting Information

Table S1 **Actors, their Civilian Targeting Index (CTI), and their Total Associated Fatalities during 2002–2007.** Separate file, for online supporting information.
(DOC)
