# Supplementary material for: Global Comparison of Warring Groups in 2002–2007: Fatalities from Targeting Civilians vs. Fighting Battles
Source: PLoS One. 2011 Sep 6;6(9):e23976. doi: 10.1371/journal.pone.0023976 (PMC3167835; doi:10.1371/journal.pone.0023976)
Supplement: Table S1 — Actors, their Civilian Targeting Index (CTI), and their Total Associated Fatalities during 2002–2007. Separate file, for online supporting information. (DOC) [file pone.0023976.s001.doc]

**Table S1. Actors, their Civilian Targeting Index (CTI), and their Total Associated Fatalities during 2002-2007.**

| **Range of Fatalities** | **State Actors** | | | | | **Non-state Actors** | | | | | |
| --- | --- | --- | --- | --- | --- | --- | --- | --- | --- | --- | --- |
|  | **Actors** | **CTI**  **(%)** | **Rank by CTI** | **Total Fatalities** | **Rank by Fatalities** | **Actors** | **Location of Incompatibility** | **CTI**  **(%)** | **Rank by CTI** | **Total Fatalities** | **Rank by Fatalities** |
| **Over 10,000** *States: 3. Non-states: 2.* | Sudan | 36.5 | 39 | 14,145 | 2 | Islamic State of Iraq (ISI or Dawlat al-’Iraq al-Islamiyya) | Iraq, Jordan | 21.6 | 50 | 11,748 | 3 |
|  | Iraq | 1.5 | 84 | 19,956 | 1 | Taleban | Afghanistan | 2.7 | 82 | 11,524 | 4 |
|  | Afghanistan | 0 | 89 | 11,214 | 5 |  |  |  |  |  |  |
| **1,000 to 9,999** *States: 22. Non-states: 26.* | Ethiopia | 70.8 | 28 | 1,357 | 46 | Front for National Integration (FNI or Forces Nationalistes et Integrationistes) | Congo | 96.1 | 26 | 1,314 | 49 |
|  | Myanmar | 48.9 | 33 | 1,398 | 42 | Janjaweed (Janjaweed only. Janjaweed with Sudan is coded under Sudan.) | CAR, Chad, Sudan | 93.5 | 27 | 3,298 | 20 |
|  | Cote D’Ivoire | 31.8 | 44 | 1,080 | 52 | Patani insurgents | Thailand | 57.2 | 31 | 1,886 | 30 |
|  | Congo | 31.4 | 45 | 1,132 | 51 | Lord's Resistance Army (LRA) | Sudan, Uganda | 40.9 | 36 | 7,439 | 11 |
|  | Indonesia | 15.6 | 57 | 1,826 | 33 | Communist Party of India-Maoist (CPI-M) | India | 30.3 | 46 | 1,391 | 43 |
|  | US (US only. US with Iraq or Afghanistan coded under those actors)* | 14.4 | 59 | 1,721 | 35 | United Self-Defense Forces of Colombia (AUC or Autodefensas Unidas de Colombia) | Colombia | 20.9 | 51 | 1,428 | 40 |
|  | Burundi | 11.3 | 64 | 2,688 | 23 | Kashmir Insurgents | India | 20.2 |  | 7,745 |  |
|  | Israel* | 11.0 | 65 | 2,944 | 22 | Hamas | Israel | 18.3 | 55 | 1,362 | 45 |
|  | Nepal | 8.4 | 71 | 9,531 | 7 | Party for the Liberation of the Hutu People-Forces for National Liberation (Palipehutu-FNL or Parti pour la Libération du Peuple Hutu-Forces Nationales de Liberation) | Burundi | 15.5 | 58 | 2,295 | 26 |
|  | Russia | 5.6 | 75 | 3,692 | 18 | Free Aceh Movement (GAM or Gerakan Aceh Merdeka) | Indonesia | 13.0 | 61 | 1,771 | 34 |
|  | India* | 1.5 | 85 | 9,413 | 8 | Communist Party of Nepal-Maoist (CPN-M) | Nepal | 10.7 | 66 | 9,815 | 6 |
|  | Somalia | 1.2 | 87 | 2,061 | 29 | Moro Islamic Liberation Front (MILF) | Philippines | 10.7 | 67 | 1,060 | 53 |
|  | Sri Lanka | 0.7 | 88 | 3,995 | 17 | Ansar al-Islam | Iraq | 8.6 | 70 | 1,374 | 44 |
|  | US/UK/Australia (US/UK/Australia only. With Iraq is coded under Iraq)* | 0 | 89 | 8,202 | 9 | Revolutionary Armed Forces of Colombia (FARC or Fuerzas Armadas Revolucionarias Colombianas) | Colombia | 8.4 | 72 | 6,219 | 12 |
|  | Colombia | 0 | 89 | 5,142 | 13 | Al-Mahdi Army | Iraq | 6.9 | 73 | 1,685 | 37 |
|  | Uganda* | 0 | 89 | 4,352 | 15 | Al-Qaida Organisation in the Islamic Maghreb (AQIM) | Algeria | 6.0 | 74 | 1,712 | 36 |
|  | Philippines | 0 | 89 | 3,159 | 21 | Justice and Equality Movement (JEM) | Sudan | 5.2 | 76 | 1,323 | 48 |
|  | Liberia | 0 | 89 | 2,116 | 27 | Al-Qaida (2002-2007 data do not include September 11, 2001 attack) | US, Saudi Arabia | 5.1 | 77 | 1,554 | 38 |
|  | Algeria | 0 | 89 | 1,846 | 31 | Chechen Republic of Ichkeria | Russia | 4.6 | 79 | 3,624 | 19 |
|  | Chad | 0 | 89 | 1,837 | 32 | Communist Party of the Philippines (CPP) | Philippines | 3.6 | 80 | 1,401 | 41 |
|  | Pakistan* | 0 | 89 | 1,518 | 39 | Liberation Tigers of Tamil Eelam (LTTE or Thamil Eelam Viduthalai Puligal) | Sri Lanka | 3.2 | 81 | 4,311 | 16 |
|  | Turkey | 0 | 89 | 1,342 | 47 | Sudanese People’s Liberation Movement/Army (SPLM/A) | Sudan | 2.2 | 83 | 2,600 | 24 |
|  |  |  |  |  |  | Sudan Liberation Movement/Army (SLM/A) | Sudan | 1.4 | 86 | 4,457 | 14 |
|  |  |  |  |  |  | Alliance for the Re-liberation of Somalia/Islamic Courts Union (ARS/UIC) | Somalia | 0 | 89 | 2,502 | 25 |
|  |  |  |  |  |  | Liberians United for Reconciliation and Democracy ( LURD) | Liberia | 0 | 89 | 2,089 | 28 |
|  |  |  |  |  |  | Kurdistan Workers' Party (PKK or Partiya Karkerên Kurdistan) | Turkey | 0 | 89 | 1,313 | 50 |
| **100 to 999** *States: 6. Non-states: 79.* | Nigeria | 50.6 | 32 | 261 | 96 | Congolese Democratic Rally-Goma (RCD-Goma or Rassemblement Congolaises pour la Démocratie-Goma) | Congo | 100 | 1 | 465 | 75 |
|  | Central African Republic (CAR) | 47 | 35 | 283 | 95 | World Hindu Council (VHP or Vishwa Hindu Parishad) | India | 100 | 1 | 401 | 82 |
|  | Thailand | 19.2 | 54 | 999 | 54 | Jemaah Islamiya | Indonesia | 100 | 1 | 202 | 107 |
|  | Haiti | 14.1 | 60 | 284 | 94 | Moroccan Islamic Combatant Group (GICM) | Spain | 100 | 1 | 191 | 109 |
|  | Angola | 10 | 69 | 590 | 64 | Jamaat Jund al-Sahaba | Iraq | 100 | 1 | 156 | 121 |
|  | Iran* | 0 | 89 | 157 | 119 | Mayi Mayi-Chinja Chinja | Congo | 100 | 1 | 137 | 125 |
|  |  |  |  |  |  | Tawhid wal Jihad | Egypt | 100 | 1 | 122 | 133 |
|  |  |  |  |  |  | Rastas | Rwanda | 100 | 1 | 121 | 134 |
|  |  |  |  |  |  | Lashkar-e-Jhangvi | Pakistan | 100 | 1 | 110 | 136 |
|  |  |  |  |  |  | Democratic Liberation Forces of Rwanda (FDLR or Forces Démocratiques de Libération du Rwanda) | Congo, Rwanda | 69.9 | 29 | 196 | 108 |
|  |  |  |  |  |  | Congolese Rally for Democracy-National/ Movement for the Liberation of Congo (RCD-N or Rassemblement Congolaises pour la Démocratie-National/MLC or Mouvement de Libération Congolais) | Congo | 67.7 | 30 | 446 | 78 |
|  |  |  |  |  |  | Armed Islamic Group (GIA or Groupe Islamique Armé) | Algeria | 48.2 | 34 | 456 | 76 |
|  |  |  |  |  |  | United Liberation Front of Assam (ULFA) | India | 40.6 | 37 | 665 | 60 |
|  |  |  |  |  |  | National Democratic Front for Bodoland (NDFB) | India | 40.2 | 38 | 291 | 93 |
|  |  |  |  |  |  | Ntsiloulous | Congo | 35.6 | 40 | 163 | 117 |
|  |  |  |  |  |  | Ivorian Movement for the Greater West (MPIGO or Mouvement Populaire Ivorian du Grand Ouest) | Cote D’Ivoire | 34.0 | 42 | 191 | 109 |
|  |  |  |  |  |  | al-Aqsa Martyrs' Brigades (AMB or Kataeb al-Shaheed al-Aqsa) | Israel | 32.0 | 43 | 387 | 83 |
|  |  |  |  |  |  | National Liberation Front of Tripura (NLFT) | India | 28.4 | 47 | 296 | 92 |
|  |  |  |  |  |  | Ogaden National Liberation Front (ONLF) | Ethiopia | 25.0 | 48 | 328 | 85 |
|  |  |  |  |  |  | Patriotic Movement of Ivory Coast (MPCI or Mouvement patriotique de la Côte d’Ivoire) | Cote D’Ivoire | 23.5 | 49 | 650 | 61 |
|  |  |  |  |  |  | National Council for the Defence of Democracy-Forces for the Defence of Democracy (CNDD-FDD or Conseil National pour la Défense de la Démocratie-Forces pour la Défense de la Démocratie) | Burundi, Congo | 19.9 | 53 | 860 | 57 |
|  |  |  |  |  |  | Abu Sayyaf Group (ASG) | Philippines | 17.0 | 56 | 946 | 56 |
|  |  |  |  |  |  | Palestinian Islamic Jihad (PIJ or Harakat al-Jihad al-Islami fi Filastin) | Israel | 12.4 | 62 | 492 | 70 |
|  |  |  |  |  |  | National Union for the Total Independence of Angola (UNITA or União Nacional para a Independência Total de Angola) | Angola | 12.1 | 63 | 471 | 73 |
|  |  |  |  |  |  | Mayi Mayi | Congo | 10.6 | 68 | 479 | 71 |
|  |  |  |  |  |  | National Congress for the Defence of the People (CNDP or Congrès National pour la Défense du Peuple) | Congo | 5.0 | 78 | 585 | 65 |
|  |  |  |  |  |  | United Front for Democratic Change (FUCD) | Chad | 0 | 89 | 979 | 55 |
|  |  |  |  |  |  | Hezbollah | Israel | 0 | 89 | 821 | 58 |
|  |  |  |  |  |  | National Redemption Front (NRF) | Sudan | 0 | 89 | 810 | 59 |
|  |  |  |  |  |  | Congolese Democratic Rally (RCD or Rassemblement Congolaises pour la Démocratie) | Congo | 0 | 89 | 649 | 62 |
|  |  |  |  |  |  | Patriotic Union of Kurdistan (PUK) | Iraq | 0 | 89 | 626 | 63 |
|  |  |  |  |  |  | Movement for the Enforcement of Islamic Laws (TNSM or Tehreek-e-Nafaz-e-Shariat-e-Mohammadi) | Pakistan | 0 | 89 | 578 | 66 |
|  |  |  |  |  |  | Alliance for the Restoration of Peace and Counter-Terrorism (ARPCT or Isbaheysiga Ladagaalanka Argagaxisadda) | Somalia | 0 | 89 | 562 | 67 |
|  |  |  |  |  |  | National Liberation Army (ELN or Ejército de Liberatión Nacional) | Colombia | 0 | 89 | 546 | 68 |
|  |  |  |  |  |  | People's Democratic Party (PDP) | Nigeria | 0 | 89 | 513 | 69 |
|  |  |  |  |  |  | All Nigeria People's Party (ANPP) | Nigeria | 0 | 89 | 478 | 72 |
|  |  |  |  |  |  | Shan State Army-South command (SSA-S) | Myanmar | 0 | 89 | 469 | 74 |
|  |  |  |  |  |  | Fatah | Israel | 0 | 89 | 452 | 77 |
|  |  |  |  |  |  | Karen National Union (KNU) | Myanmar | 0 | 89 | 425 | 79 |
|  |  |  |  |  |  | Movement for Democracy and Justice in Chad (MDJT or Mouvement pour la Démocratie et la Justice au Tchad) | Chad | 0 | 89 | 418 | 80 |
|  |  |  |  |  |  | Union Force for Democracy and Development (UFDD or Union des Forces pour la Démocratie et le Développement) | Chad | 0 | 89 | 408 | 81 |
|  |  |  |  |  |  | National Socialist Council of Nagaland-Khaplang faction (NSCN-K) | India | 0 | 89 | 360 | 84 |
|  |  |  |  |  |  | Reformation and Jihad Front (RJF) | Iraq | 0 | 89 | 324 | 86 |
|  |  |  |  |  |  | Islamic Movement of Uzbekistan (IMU) | Pakistan | 0 | 89 | 319 | 87 |
|  |  |  |  |  |  | Lashkha of Wazir tribe | Pakistan | 0 | 89 | 319 | 88 |
|  |  |  |  |  |  | Rahanweyn Resistance Army (RRA) | Somalia | 0 | 89 | 310 | 89 |
|  |  |  |  |  |  | Rahanweyn Resistance Army-Madobe and Habsade faction (RRA-MH) | Somalia | 0 | 89 | 310 | 89 |
|  |  |  |  |  |  | National Democratic Alliance (NDA) | Sudan | 0 | 89 | 300 | 91 |
|  |  |  |  |  |  | Sudan Liberation Movement/Army-Minni Minawi faction (SLM/A-MM) | Sudan | 0 | 89 | 236 | 97 |
|  |  |  |  |  |  | Forces of Rashid Abdul Dostum | Afghanistan | 0 | 89 | 225 | 98 |
|  |  |  |  |  |  | Forces of Ustad Mohammad Atta | Afghanistan | 0 | 89 | 225 | 98 |
|  |  |  |  |  |  | People’s War Group (PWG) | India | 0 | 89 | 224 | 100 |
|  |  |  |  |  |  | Congolese Rally for Democracy-Patrick Masunzu faction (RCD-PM or Rassemblement Congolaise pour la Démocratie-Patrick Mazunsu faction) | Congo | 0 | 89 | 221 | 101 |
|  |  |  |  |  |  | Gulf Cartel | Mexico | 0 | 89 | 218 | 102 |
|  |  |  |  |  |  | Sinaloa Cartel | Mexico | 0 | 89 | 218 | 102 |
|  |  |  |  |  |  | New Forces (FN or Forces Nouvelles) | Cote D’Ivoire | 0 | 89 | 217 | 104 |
|  |  |  |  |  |  | Baluch Ittehad | Pakistan | 0 | 89 | 214 | 105 |
|  |  |  |  |  |  | Liberation Tigers of Tamil Eelam-Karuna faction (LTTE-K) | Sri Lanka | 0 | 89 | 208 | 106 |
|  |  |  |  |  |  | National Front for the Liberation of Haiti (FLRN or Front pour la Liberación et la Reconstruction Nationales) | Haiti | 0 | 89 | 183 | 111 |
|  |  |  |  |  |  | United Wa State Army (UWSA) | Myanmar | 0 | 89 | 182 | 112 |
|  |  |  |  |  |  | National Socialist Council of Nagaland-Isaac-Muivah faction (NSCN-IM) | India | 0 | 89 | 175 | 113 |
|  |  |  |  |  |  | United National Liberation Front (UNLF) | India | 0 | 89 | 175 | 113 |
|  |  |  |  |  |  | Alliance of Democratic Forces (ADF) | Uganda | 0 | 89 | 167 | 115 |
|  |  |  |  |  |  | Baluchistan Liberation Army (BLA) | Pakistan | 0 | 89 | 165 | 116 |
|  |  |  |  |  |  | Forces of Amanullah Khan | Afghanistan | 0 | 89 | 158 | 118 |
|  |  |  |  |  |  | Somali Salvation Democratic Front (SSDF) | Somalia, Sudan | 0 | 89 | 157 | 119 |
|  |  |  |  |  |  | Oromo Liberation Front (OLF) | Ethiopia | 0 | 89 | 150 | 122 |
|  |  |  |  |  |  | Congolese Democratic Rally-Liberation Movement (RCD-ML or Rassemblement Congolaises pour la Démocratie-Mouvement de Liberation) | Congo | 0 | 89 | 144 | 123 |
|  |  |  |  |  |  | Niger Delta People’s Volunteer Force (NDPVF) | Nigeria | 0 | 89 | 140 | 124 |
|  |  |  |  |  |  | New Forces-Ibrahim Coulibaly faction (FN-IC or Forces Nouvelles-Ibrahim Coulibaly faction) | Cote D’Ivoire | 0 | 89 | 134 | 126 |
|  |  |  |  |  |  | Maoist Communist Centre (MCC) | India | 0 | 89 | 134 | 126 |
|  |  |  |  |  |  | United Somali Congress/Somali Salvation Alliance (USC/SSA) | Somalia | 0 | 89 | 134 | 126 |
|  |  |  |  |  |  | United Somalia Congress/Somali Salvation Alliance-Omar Mohamed Mohamud-Finish faction (USC/SSA-F) | Somalia | 0 | 89 | 134 | 126 |
|  |  |  |  |  |  | Forces of Ismail Khan | Afghanistan | 0 | 89 | 128 | 130 |
|  |  |  |  |  |  | Movement of the Democratic Forces of the Casamance-Northern Front Magne Diémé faction (MFDC Front Nord-MD or Mouvement des Forces Démocratiques de Casamance-Front Nord Magne Diémé) | Senegal | 0 | 89 | 124 | 131 |
|  |  |  |  |  |  | Movement of the Democratic Forces of the Casamance-Salif Sadio faction (MFDC-S or Mouvement des Forces Démocratiques de Casamance-Sadio) | Senegal | 0 | 89 | 124 | 131 |
|  |  |  |  |  |  | Kingdom of Kongo (BDK or Bundu dia Kongo) | Congo | 0 | 89 | 116 | 135 |
|  |  |  |  |  |  | The Free Life Party of Kurdistan (PJAK or Parti Jiyani Azadi Kurdistan) | Iran | 0 | 89 | 106 | 137 |
|  |  |  |  |  |  | Forces of Francois Bozize | Central African Republic | 0 | 89 | 105 | 138 |
| **Under 100** *States: 12. Non-states: 76.* | Laos | 100 | 1 | 73 | 145 | Abu-Hafs al-Masri Brigades | Turkey | 100 | 1 | 62 | 151 |
|  | Guinea | 100 | 1 | 45 | 167 | Taleban Movement of Pakistan (TTP or Tehrik-i-Taliban Pakistan) | Pakistan | 100 | 1 | 54 | 158 |
|  | Egypt | 100 | 1 | 36 | 184 | Salafia Jihadia | Morocco | 100 | 1 | 45 | 167 |
|  | Brazil | 100 | 1 | 34 | 189 | All Tripura Tiger Force (ATTF) | India | 100 | 1 | 43 | 175 |
|  | Niger | 0 | 89 | 81 | 142 | Gazotan Murdash | Russia | 100 | 1 | 41 | 176 |
|  | Rwanda | 0 | 89 | 59 | 154 | Mungiki | Kenya | 100 | 1 | 38 | 181 |
|  | Eritrea | 0 | 89 | 57 | 156 | Bakassi boys | Nigeria | 100 | 1 | 32 | 195 |
|  | Senegal | 0 | 89 | 40 | 177 | Students' Islamic Movement of India (SIMI) | India | 100 | 1 | 31 | 200 |
|  | Uzbekistan | 0 | 89 | 35 | 185 | Congolese Rally for Democracy-Mutineer faction (RCD-Mutineer faction or Rassemblement Congolaise pour la Democratie-Mutineer) | Congo | 100 | 1 | 30 | 201 |
|  | Peru | 0 | 89 | 32 | 195 | Mara Salvatrucha (Honduras) | Honduras | 100 | 1 | 28 | 207 |
|  | Georgia | 0 | 89 | 27 | 212 | Sabaot Land Defence Force (SLDF or Sabaot Land Defence Force) | Kenya | 100 | 1 | 28 | 207 |
|  | Azerbaijan | 0 | 89 | 26 | 217 | Movement of the Democratic Forces of the Casamance-Northern Front (MFDC-FN or Mouvement des Forces Démocratiques de Casamance-Front Nord) | Senegal | 100 | 1 | 25 | 220 |
|  |  |  |  |  |  | People's Armed Forces of Congo (FAPC or Forces Armées du Peuple Congolais) | Congo | 35.4 | 41 | 79 | 144 |
|  |  |  |  |  |  | Somali Reconciliation and Restoration Council (SRRC) | Somalia | 0 | 89 | 96 | 139 |
|  |  |  |  |  |  | Front for the Liberation of the Enclave of Cabinda-Armed Forces of Cabinda (FLEC-FAC or Frente da Libertação do Enclave de Cabinda-Forças Armadas de Cabinda) | Angola | 0 | 89 | 92 | 140 |
|  |  |  |  |  |  | Sudan Liberation Movement/Army-Unity (SLM/A-Unity) | Sudan | 0 | 89 | 86 | 141 |
|  |  |  |  |  |  | Niger Movement for Justice (MNJ or Mouvement des Nigériens pour la Justice) | Niger | 0 | 89 | 81 | 142 |
|  |  |  |  |  |  | Arrow Boys | Uganda | 0 | 89 | 71 | 146 |
|  |  |  |  |  |  | Somali National Front-Ali Dheere and Rer Ahmad subclans (SNF-ADRA) | Somalia | 0 | 89 | 69 | 147 |
|  |  |  |  |  |  | Somali National Front-Hawarsame Rer Hasan and Habar Ya'qub subclans (SNF-HRHHY) | Somalia | 0 | 89 | 69 | 147 |
|  |  |  |  |  |  | People's Liberation Army (PLA) | India | 0 | 89 | 67 | 149 |
|  |  |  |  |  |  | Niger Delta Vigilantes (NDV) | Nigeria | 0 | 89 | 63 | 150 |
|  |  |  |  |  |  | Jubba Valley Alliance (JVA) | Somalia | 0 | 89 | 62 | 151 |
|  |  |  |  |  |  | OP Lavalas | Haiti | 0 | 89 | 61 | 153 |
|  |  |  |  |  |  | Movement for Justice and Peace (MJP or Mouvement pour la Justice et la Paix) | Cote D’Ivoire | 0 | 89 | 59 | 154 |
|  |  |  |  |  |  | Eritrean Islamic Jihad Movement-Abu Suhail faction (EIJM-AS or Harakat al Jihad al Islami-Abu Suhail faction) | Eritrea | 0 | 89 | 57 | 156 |
|  |  |  |  |  |  | Mara 18 | Guatemala | 0 | 89 | 54 | 158 |
|  |  |  |  |  |  | Mara Salvatrucha (Guatemala) | Guatemala | 0 | 89 | 54 | 158 |
|  |  |  |  |  |  | Ahlul Sunnah Jamaa | Nigeria | 0 | 89 | 52 | 161 |
|  |  |  |  |  |  | Forces of Abdullahi Yusuf | Somalia | 0 | 89 | 52 | 161 |
|  |  |  |  |  |  | Forces of Jama Ali Jama | Somalia | 0 | 89 | 52 | 161 |
|  |  |  |  |  |  | Moro National Liberation Front-Nur Misauri faction (MNLF-NM) | Philippines | 0 | 89 | 52 | 161 |
|  |  |  |  |  |  | Jondullah | Iran | 0 | 89 | 51 | 165 |
|  |  |  |  |  |  | Palipehutu-FNL-LP | Burundi | 0 | 89 | 50 | 166 |
|  |  |  |  |  |  | Popular Resistance Committees (PRC) | Israel | 0 | 89 | 45 | 167 |
|  |  |  |  |  |  | Congolese Democratic Rally-Kisangani-Liberation Movement (RCD-K-ML or Rassemblement Congolaises pour la Démocratie-Kisangani-Mouvement de Liberation) | Congo | 0 | 89 | 45 | 167 |
|  |  |  |  |  |  | Congolese Rally for Democracy-National (RCD-N or Rassemblement Congolaises pour la Démocratie-National) | Congo | 0 | 89 | 45 | 167 |
|  |  |  |  |  |  | Union of Democratic Forces for Unity (UFDR or Union des Forces Démocratiques pour le Rassemblement) | Central African Republic | 0 | 89 | 45 | 167 |
|  |  |  |  |  |  | Janjaweed-Bin Kulaib faction | Sudan | 0 | 89 | 44 | 173 |
|  |  |  |  |  |  | Janjaweed-Moro faction | Sudan | 0 | 89 | 44 | 173 |
|  |  |  |  |  |  | Movement of the Democratic Forces of the Casamance (MFDC or Mouvement des Forces Démocratiques de Casamance) | Senegal | 0 | 89 | 40 | 177 |
|  |  |  |  |  |  | May 23 Democratic Alliance for Change-Ibrahim Bahanga faction (ATNMC or Alliance Démocratique du 23 Mai pour le Changement-Ibrahim Bahanga faction) | Mali | 0 | 89 | 39 | 179 |
|  |  |  |  |  |  | Mali | Mali | 0 | 89 | 39 | 179 |
|  |  |  |  |  |  | Popular Defence Force (PDF) | Sudan | 0 | 89 | 38 | 181 |
|  |  |  |  |  |  | Palestinian National Authority (PNA) | Israel | 0 | 89 | 38 | 181 |
|  |  |  |  |  |  | Alliance for Democracy (AD) | Nigeria | 0 | 89 | 35 | 185 |
|  |  |  |  |  |  | Jihad Islamic Group (JIG) | Uzbekistan | 0 | 89 | 35 | 185 |
|  |  |  |  |  |  | Karenni National Progressive Party (KNPP) | Myanmar | 0 | 89 | 35 | 185 |
|  |  |  |  |  |  | Ansaar ul-Islam | Pakistan | 0 | 89 | 34 | 189 |
|  |  |  |  |  |  | Lashkar-e-Islam | Pakistan | 0 | 89 | 34 | 189 |
|  |  |  |  |  |  | Puntland state of Somalia | Somalia | 0 | 89 | 34 | 189 |
|  |  |  |  |  |  | Republic of Somaliland | Somalia | 0 | 89 | 34 | 189 |
|  |  |  |  |  |  | Democratic Karen Buddhist Army (DKBA or Democratic Karen Buddhist Army) | Myanmar | 0 | 89 | 33 | 194 |
|  |  |  |  |  |  | Madhesi People's Rights Forum (MJF or Madhesi Jana Adhikar Forum) | Nepal | 0 | 89 | 32 | 195 |
|  |  |  |  |  |  | Rally of Democratic Forces (RAFD or Rassemblement des Forces Démocratiques) | Chad | 0 | 89 | 32 | 195 |
|  |  |  |  |  |  | Sendero Luminoso | Peru | 0 | 89 | 32 | 195 |
|  |  |  |  |  |  | Comando Vermelho | Brazil | 0 | 89 | 30 | 201 |
|  |  |  |  |  |  | Forces of Arbab Basir | Afghanistan | 0 | 89 | 30 | 201 |
|  |  |  |  |  |  | Forces of the Caucasus Emirate | Russia | 0 | 89 | 30 | 201 |
|  |  |  |  |  |  | Tercer Comando | Brazil | 0 | 89 | 30 | 201 |
|  |  |  |  |  |  | Maoist Communist Party (MKP or Maoist Komünist Partisi) | Turkey | 0 | 89 | 29 | 206 |
|  |  |  |  |  |  | Movement for the Liberation of Western Ivory Coast (MILOCI or Mouvement pour la Liberation de l'Ouest de la Côte d'Ivoire) | Cote D’Ivoire | 0 | 89 | 28 | 207 |
|  |  |  |  |  |  | Southern Somalia National Movement (SSNM) | Somalia | 0 | 89 | 28 | 207 |
|  |  |  |  |  |  | United Somali Congress/Somali National Alliance (USC/SNA) | Somalia | 0 | 89 | 28 | 207 |
|  |  |  |  |  |  | 14-party Alliance | Bangladesh | 0 | 89 | 27 | 212 |
|  |  |  |  |  |  | Bangladesh National Party Alliance (BNP Party Alliance) | Bangladesh | 0 | 89 | 27 | 212 |
|  |  |  |  |  |  | Movement for Democracy in Liberia (MODEL) | Liberia | 0 | 89 | 27 | 212 |
|  |  |  |  |  |  | Republic of South Ossetia | Georgia | 0 | 89 | 27 | 212 |
|  |  |  |  |  |  | People’s Liberation Army (EPL or Ejército Popular de Liberación) | Colombia | 0 | 89 | 26 | 217 |
|  |  |  |  |  |  | Republic of Nagorno-Karabakh | Azerbaijan | 0 | 89 | 26 | 217 |
|  |  |  |  |  |  | 1920 Revolution Brigades | Iraq | 0 | 89 | 25 | 220 |
|  |  |  |  |  |  | Front for the Liberation of the Enclave of Cabinda-Renewed (FLEC-R or Frente da Libertação do Enclave de Cabinda-Renovada) | Angola | 0 | 89 | 25 | 220 |
|  |  |  |  |  |  | Forces of Abdul Rahman Khan | Afghanistan | 0 | 89 | 25 | 220 |
|  |  |  |  |  |  | Forces of Amanullah | Afghanistan | 0 | 89 | 25 | 220 |
|  |  |  |  |  |  | Jubba Valley Alliance faction (JVA faction) | Somalia | 0 | 89 | 25 | 220 |
|  |  |  |  |  |  | Moro National Liberation Front-Habier Malik faction (MNLF-HM) | Philippines | 0 | 89 | 25 | 220 |
| **Average** *States: 43. Non-states: 183.* |  | **19.3** (95% CI: 10 to 29) |  | **2809** (95% CI: 1495 to 4123) |  |  |  | **17.3** (95% CI: 12 to 22) |  | **708** (95% CI: 452 to 963) |  |

** Locations of armed conflicts for state actors are their own territories except the US (Afghanistan/Iraq/Pakistan), Israel (Israel/Lebanon), India (India/Pakistan/Myanmar), US/UK/Australia (US/UK/Australia/Iraq), Uganda (Uganda/Congo/Sudan), Pakistan (Pakistan/India) and Iran (Iran/Iraq).*
